# Role of Immune Cell Diversity and Heterogeneity in Corneal Graft Survival: A Systematic Review and Meta-Analysis

**DOI:** 10.3390/jcm10204667

**Published:** 2021-10-12

**Authors:** Jun Zhu, Takenori Inomata, Antonio Di Zazzo, Koji Kitazawa, Yuichi Okumura, Marco Coassin, Pier Luigi Surico, Kenta Fujio, Ai Yanagawa, Maria Miura, Yasutsugu Akasaki, Keiichi Fujimoto, Ken Nagino, Akie Midorikawa-Inomata, Kunihiko Hirosawa, Mizu Kuwahara, Tianxiang Huang, Hurramhon Shokirova, Atsuko Eguchi, Akira Murakami

**Affiliations:** 1 Department of Ophthalmology, Juntendo University Graduate School of Medicine, Tokyo 1130033, Japan; j.zhu.gx@juntendo.ac.jp (J.Z.); y-okumura@juntendo.ac.jp (Y.O.); k.fujio.zz@juntendo.ac.jp (K.F.); maria-k@juntendo.ac.jp (M.M.); y-akasaki@juntendo.ac.jp (Y.A.); k-hirosawa@juntendo.ac.jp (K.H.); mz-ohno@juntendo.ac.jp (M.K.); h.tianxiang.zb@juntendo.ac.jp (T.H.); h-shokirova@juntendo.ac.jp (H.S.); amurak@juntendo.ac.jp (A.M.); 2 Department of Ophthalmology, Subei People’s Hospital of Jiangsu Province, Yangzhou 225001, China; 3 Department of Strategic Operating Room Management and Improvement, Juntendo University Graduate School of Medicine, Tokyo 1130033, Japan; 4 Department of Hospital Administration, Juntendo University Graduate School of Medicine, Tokyo 1130033, Japan; k-nagino@juntendo.ac.jp (K.N.); ak-inomata@juntendo.ac.jp (A.M.-I.); a-eguchi@juntendo.ac.jp (A.E.); 5 Department of Digital Medicine, Juntendo University Graduate School of Medicine, Tokyo 1130033, Japan; miya-ai@juntendo.ac.jp (A.Y.); k-fujimoto@juntendo.ac.jp (K.F.); 6 Department of Ophthalmology, Faculty of Medicine, Juntendo University, Tokyo 1130033, Japan; 7 Ophthalmology Complex Operative Unit, Campus Bio-Medico University Hospital, 00128 Rome, Italy; antoniodizazzo@gmail.com (A.D.Z.); m.coassin@unicampus.it (M.C.); pierluigi.surico@unicampus.it (P.L.S.); 8 Department of Ophthalmology, Kyoto Prefectural University of Medicine, Kyoto 6020841, Japan; kkitazaw@koto.kpu-m.ac.jp; 9 Buck Institute for Research on Aging, Novato, CA 94945, USA

**Keywords:** corneal transplantation, immune cell, diversity, heterogeneity, innate immunity, adaptive immunity, systematic review, meta-analysis

## Abstract

Corneal transplantation is one of the most successful forms of solid organ transplantation; however, immune rejection is still a major cause of corneal graft failure. Both innate and adaptive immunity play a significant role in allograft tolerance. Therefore, immune cells, cytokines, and signal-transduction pathways are critical therapeutic targets. In this analysis, we aimed to review the current literature on various immunotherapeutic approaches for corneal-allograft rejection using the PubMed, EMBASE, Web of Science, Cochrane, and China National Knowledge Infrastructure. Retrievable data for meta-analysis were screened and assessed. The review, which evaluated multiple immunotherapeutic approaches to prevent corneal allograft rejection, showed extensive involvement of innate and adaptive immunity components. Understanding the contribution of this immune diversity to the ocular surface is critical for ensuring corneal allograft survival.

## 1. Introduction

Corneal transplantation has been proven to be the most effective therapy for corneal disorders such as opacity, keratoconus, corneal degeneration, scarring due to keratitis, trauma, and any physio-pathological changes on the ocular surface [1,2,3]. In addition to the appropriate examination of the characteristics of the donor and the tissue [4], immune privilege plays an important role in the success of corneal transplantation procedures [5,6].

Corneal immune and angiogenic privilege [7] is crucial to the success of corneal transplantation and is mainly dependent on resident heterogeneous immune cells, including dendritic cells (DCs), Langerhans cells (LCs), mast cells, macrophages, T lymphocytes, and regulatory T cells [8,9,10,11]. Additionally, the systemic immune response in certain autoimmune diseases (such as Sjögren’s syndrome, systemic lupus erythematosus, and rheumatoid arthritis) affects the homeostasis of the ocular surface immune microenvironment, leading to the loss of corneal avascularity [12,13]. The underlying mechanism involves various components of the immune system [14]. Consequently, immunological approaches have been introduced to improve corneal graft survival. Conventional prophylaxis, including topical or systemic medications such as corticosteroids, cyclosporine A, and tacrolimus, has been proven to be successful [15,16,17]. Although immunosuppressive drugs show promising effectiveness, their side effects such as cataracts, susceptibility to infection, and glaucoma cannot be disregarded [18,19]. Several studies have been conducted on immunotherapy for corneal transplantation focusing on immune checkpoint inhibitors, human leukocyte antigen (HLA)-matching strategy, and immunomodulatory cytokines [20,21,22]. These studies give extraordinary contributions to the management of corneal graft rejections.

In this review, various immunotherapies for corneal transplantation were assessed to explore the effects on different immune cell populations, relative cytokines, and signaling pathways involved in graft survival. The results from this study could represent a guide for further independent studies focusing on alternative immunotherapy strategies for corneal transplantation.

## 2. Materials and Methods

### 2.1. Database Retrieval and Search Strategy

Electronic bibliographic databases including PubMed, EMBASE, Cochrane, Web of Science, and China National Knowledge Infrastructure were used to collect published research papers (from January 1988 to December 2020), by combining the genetic terms. The final formula was as follows: [(corneal transplant) OR (corneal transplantation) OR (corneal grafts) OR (keratoplasty) OR (corneal allografts) OR (corneal graft survival) OR (corneal allograft survival) OR (keratoplasty survival)] AND [(innate) OR (adaptive) OR (immune) OR (innate immune) OR (adaptive immune)]. The design of this study followed the guidelines of the Preferred Reporting Items for Systematic Reviews and Meta-Analyses (PRISMA) protocols [23]. The published languages were limited to English, Chinese, and German. The search results were screened for suitable topics and full articles accessible for systematic review. Full-text articles containing integral data for meta-analysis were collected. The study inclusion and exclusion criteria are presented in Table 1. Search results were compiled using EndNote X9.3.2 (Clarivate Analytics, Philadelphia, PA, USA). In keeping with the quality standards for reporting systematic reviews and meta-analyses of observational studies [24], two independent researchers (J.Z. and T.I.) screened the retrieved articles. The same investigators independently assessed the full text of the records that were deemed eligible in consensus.

### 2.2. Data Extraction

Two independent reviewers (J.Z. and T.I.) extracted data from each eligible study using a standardized data-extraction sheet and subsequently cross-checked the results. Disagreements between the reviewers regarding the extracted data were resolved through discussion with a third reviewer (Y.O.). The following data were extracted: author’s first name, date of publication, details of intervention in the study and control groups, sample size, mean survival or rejection days and standard deviation (SD), follow-up period and main results, and survival or rejection ratio of the mouse or rat corneal allografts. The unit of analysis was corneal allograft.

### 2.3. Statistical Analysis

Analysis was performed using OpenMetaAnalyst version 12.11.14 (available online: http://www.cebm.brown.edu/openmeta/, accessed on 6 October 2021) [25]. The study weight was calculated using the Mantel–Haenszel method. Statistical heterogeneity was assessed using Cochran’s Q and *I*^2^ tests. *I*^2^ represents the percentage of total variation across trials, which accounts for heterogeneity. As there was no heterogeneity (*I*^2^ < 50%) among the studies, a fixed-effects model was applied. When *I*^2^ was < 50%, a random-effects analysis was performed.

## 3. Results

### 3.1. Study Characteristics

**Figure 1:**
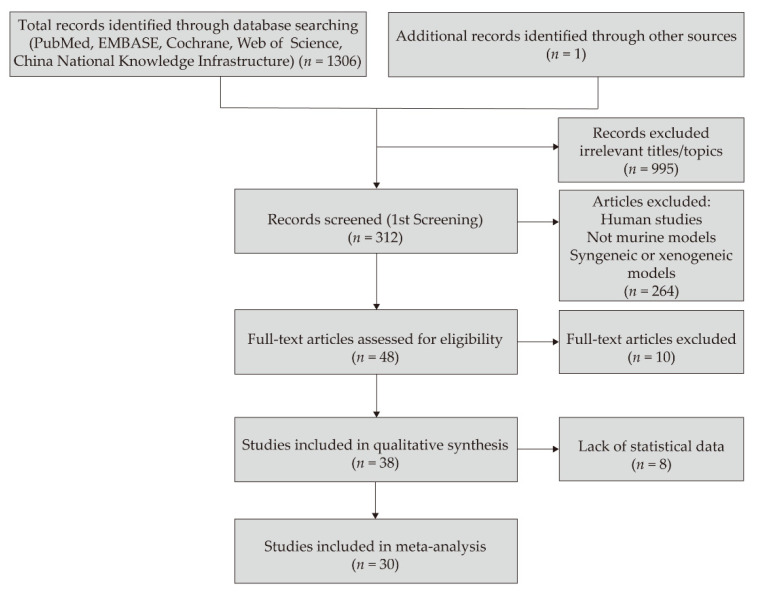
Flow chart of the studies selected for meta-analysis.

The articles included in this systematic review were published between 1 January 1988, and 31 December 2020. In total, 1307 studies were selected according to the search strategy, 995 studies were excluded owing to irrelevant titles or topics, and 264 studies with human subjects or other animal models were excluded. Thirty articles were finally included in the meta-analysis (Figure 1). Of these, 11 articles were from the United States [26,27,28,29,30,31,32,33,34,35,36], 10 from China [37,38,39,40,41,42,43,44,45,46], seven from Germany [47,48,49,50,51,52,53], and one each from France [54] and Portugal [55]. Altogether, 845 corneal allografts were identified in these studies (Table 2). Twelve studies used a rat allograft model, whereas eighteen studies used a mouse allograft model. Fifteen studies reported the mean survival or rejection days, one study reported median survival days, all thirty studies reported the survival or rejection rate, and one study reported immunoreaction days.

### 3.2. Immune Cells, Cytokines, and Pathways Associated with Allograft Rejection and Survival

The 30 studies selected for meta-analysis covered mainstream research directions for immune therapy for corneal transplantation. The number of research papers in descending order were as follows: anti-VEGF therapy (6) > DCs and LCs (6: 3 for each) > IL-1RA (4), CLTA-4 Ig therapy (4) > anti-IL-17 antibody (2), IL-10 (2), anti-CD154 antibody (2), CCR7-CCL19 blockade (2), and regulatory T cells (2). Among these immunotherapies, immature dendritic cell intervention presented the longest mean difference in survival time by 14.61 days, and IL-17 had a protective role with a mean difference of 10.98 days. Regulatory T cells also demonstrated a strong promotion for graft privilege with a mean difference of 9.42 days.

#### 3.2.1. T-Cell Subsets in Corneal-Allograft Rejection

The role of CD4^+^ and CD8^+^ T cells in alloantigen recognition is implied in the initiation of rejection [56,57]. In this review, the respective contributions of these two cell subsets in murine corneal-allograft rejection were investigated. Six studies [58,59,60,61,62,63] used anti-CD4 antibodies in murine corneal transplantation. While four studies showed that systemic or local administration of anti-CD4 antibodies could reduce the rejection rate, two of these studies [59,61] reported that anti-CD8 antibody administration did not reduce the rejection rate of corneal allografts. Two other studies [64,65] examined the role of CD8^+^ T cells in allograft rejection and found that CD8^+^ T cell-mediated rejection had a slow tempo and CD8^+^ T cells were not essential in promoting corneal-graft rejection. Together, these results showed that CD4^+^ T cells play a major role in allograft rejection.

#### 3.2.2. Dendritic Cell Heterogeneity in Different Roles of Corneal Allograft Tolerance

**Figure 2:**
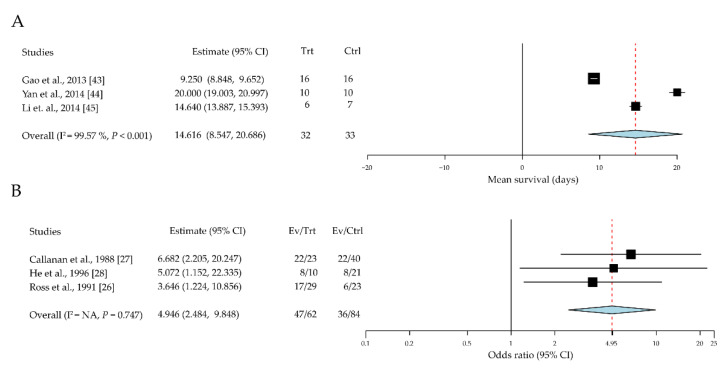
The role of the dendritic cells in allograft survival. (**A**) Immature dendritic cells promote allograft survival; (**B**) Langerhans cells increase the allograft rejection rate. CI, confidence intervals; EV, event; Trt, treatment; Ctrl, control.

DCs are potent antigen-presenting cells (APCs) with various subsets and different states. Immature DC attributes were evaluated in relation to corneal allograft tolerance in a total of 65 eyes across three studies [43,44,45]. Given immature DC intervention, the mean difference in survival time was significantly prolonged by 14.61 days (95% CI, 8.55 to 20.69; *P* < 0.001) (Figure 2A). The role of LCs, a distinct population of DCs, in corneal-allograft rejection was also evaluated; three papers with 146 cases were reviewed [26,27,28]. Compared with the control grafts (treated with immature DCs), those with donor-derived LCs exhibited a significantly increased rejection rate (odds ratio (OR) = 4.94 (95% CI, 2.48 to 9.84)) (Figure 2B).

#### 3.2.3. Macrophages Contribute to the Immunopathogenesis of Corneal-Graft Rejection

In addition to the predominant effect of T lymphocytes on allograft rejection, the involvement of macrophages in grafted corneas was reviewed. Six studies [66,67,68,69,70,71] on the effects of macrophages on corneal-allograft survival were included; their results showed the increased number of macrophages and the production of Th1 cytokines, interferon-γ (IFN-γ), interleukin-2 (IL-2), IL-12, IL-1, tumor necrosis factor-alpha (TNF-α), C-C motif chemokine ligand 3 (CCL3), and inducible nitric oxide synthase (iNOS), which were observed in rejected grafts compared to controls. Macrophages play a role in the early phase of corneal-allograft rejection. Maruyama et al. [66] found that CD11b+ macrophages are critical for the development of inflammation-dependent lymphangiogenesis in the eye. Using macrophage depletion or CD11b-/- or F4/80-/- mouse models could lead to fewer lymphatic vessels and less lymphangiogenesis, providing immune privilege to the grafts. Yamada et al. [71] reported that enhanced graft acceptance might be attributed to the suppression of alloantigen-induced Th1 polarization through the induction of macrophages with reduced intracellular glutathione levels. Together, these results suggest that macrophages are non-negligible components in the immunopathogenesis of corneal graft rejection.

#### 3.2.4. Cytokine Diversity in the Regulation of Corneal-Allograft Rejection

**Figure 3:**
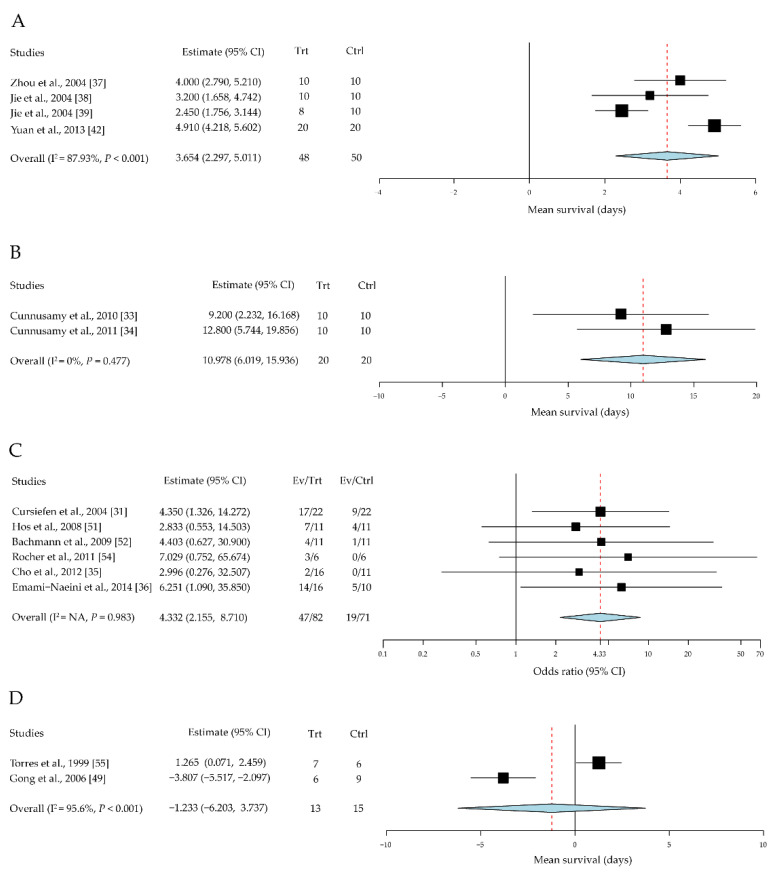
Effects of cytokines in immunotherapies for corneal allografts. (**A**) IL-1RA promotes allograft survival. (**B**) Anti-IL-17 antibody increased allograft rejection. (**C**) Anti-VEGF improves allograft survival rate. (**D**) Local administration of IL-10 did not improve allograft survival. CI, confidence intervals; EV, event; Trt, treatment; Ctrl, control.

Although numerous immune cells and their cytokine production are involved in the corneal-allograft immune reaction, few studies have generated statistical data for meta-analysis under a unified standard assessment. The representative data analysis is as follows: four studies with a total of 98 cases were included in the assessment of the effect of IL-1 receptor antagonist (IL-1RA) on corneal allografts [37,38,39,42]. The mean survival time was significantly prolonged in the IL-1RA intervention groups compared to the control group, with a mean difference of 3.65 days (95% CI, 2.30 to 5.01, *P* < 0.001) (Figure 3A).

Two studies with a total of 40 cases were included in the assessment of the effect of IL-17 on corneal allografts [33,34]. The mean rejection days were significantly reduced in the anti-IL-17 antibody intervention groups compared to the control group, with a mean difference of 10.98 days (95% CI, 6.01 to 15.94; *P* < 0.001) (Figure 3B). It is suggested that IL-17 may contribute to the immune privilege of corneal allografts. 

Six studies with a total of 153 cases were included in the assessment of the effect of vascular endothelial growth factor (VEGF) on corneal allografts [31,35,36,51,52,54]. Allograft survival rates were significantly higher in the anti-VEGF intervention groups compared to the control group, with an OR of 4.33 (95% CI, 2.16 to 8.71) (Figure 3C).

Two studies with a total of 28 cases were included in the assessment of the effect of IL-10 on corneal allografts [50,55]. No significant difference was found in the intervention groups compared to the control group. The standardized mean difference was 1.23 days (95% CI, −6.20 to 3.73, *P* = 0.627) (Figure 3D).

#### 3.2.5. Co-Stimulatory Pathways in Corneal-Allograft Survival

**Figure 4:**
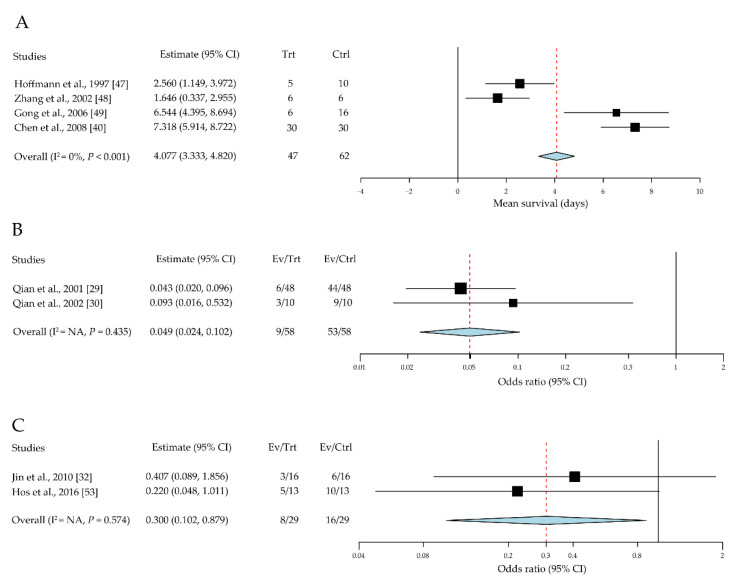
Blockage of co-stimulatory pathways influences corneal-allograft survival. (**A**) CLTA-4 Ig promotes allograft survival. (**B**) Anti-CD154 antibody increases the allograft survival rate. (**C**) CCR7-CCL19 blockade improves the allograft survival rate. CI, confidence intervals; EV, event; Trt, treatment; Ctrl, control.

In addition to the classic T-cell receptor (TCR) and major histocompatibility complex (MHC) interactions that initiate T cell activation, we investigated the function of several representative costimulatory signaling pathways in corneal-allograft survival [40,47,48,49]. For the B7-CTLA-4 interaction, we reviewed four studies including 109 cases [40,47,48,49]. The mean survival time was significantly higher in the CLTA-4 Ig intervention groups than in the control group, with a mean difference of 4.08 days (95% CI, 3.33 to 4.82, *P* < 0.001) (Figure 4A).

For the CD40-CD154 pathway, we reviewed two studies including 116 cases [29,30]. The survival rates were significantly higher in the anti-CD154 antibody intervention groups than in the control group (OR = 0.05; 95% CI, 0.03 to 0.10; *P* < 0.001) (Figure 4B).

For the C-C chemokine receptor type 7 (CCR7)-CC-chemokine ligand 19 (CCL19) pathway, we reviewed two studies including 58 cases [32,53]. The survival rates were significantly higher when CCR7-CCL19 was blocked using CCL19 Ig or CCR7-/- mouse models than the survival rate in the control group (OR = 0.30; 95% CI, 0.10 to 0.88; *P* = 0.028) (Figure 4C).

#### 3.2.6. Regulatory T Cells (Tregs) Promote Corneal Allograft Survival

**Figure 5:**
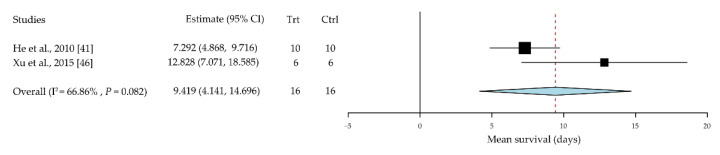
Regulatory T cells prolong corneal allograft survival. CI, confidence intervals; EV, event; Trt, treatment; Ctrl, control.

Tregs are a subset of CD4^+^ T cells with highly expressed CD4^+^CD25^+^ surface markers [72]; numerous studies have reported their immunosuppressive function in organ transplantation. In this review, two papers including thirty-two cases were assessed [41,46]. The mean survival time was significantly prolonged in the Treg intervention groups compared with that in the PBS control group, with a standard mean difference of 9.42 days (95% CI, 4.14 to 14.70; *P* < 0.001) (Figure 5).

## 4. Discussion

Immune privilege is critical to the survival of corneal transplants and includes lymphangiogenic and hemangiogenic privilege, which prevents blood and lymphatic vessels from invading the cornea. This privilege maintains the transparency of corneal tissue, which provides visual functions. During the inflammation process, inflammatory cells and their products such as cytokines and growth factors could be delivered through the blood and lymphatic vessels, leading to the immune rejection.

The process of corneal-allograft survival requires a delicate balance between immune and inflammatory reactions to provide the allografts with relative immune privilege. 

T lymphocytes play a central role in the adaptive immune response and thus strongly impact the outcome of allografts [73]. The CD4^+^ and CD8^+^ T cell were designated for allorecognition in corneal transplantation [56]. CD4^+^ T cells have been shown to play a pivotal role in corneal-allograft rejection. In a clinical report, rapamycin, an inhibitor of a serine-threonine protein kinase, mammalian target of rapamycin complex-1, could inhibit effector T cell proliferation and activation; it was found to prevent 78% rejection in the first year during the management of high-risk transplant patients [18].

As major components of the innate immune system, DCs, neutrophils, macrophages, and natural killer cells are essential for protecting against pathogens and repairing tissue damage [74]. DCs also play an important role in the innate detection of pathogens and activation of the adaptive immune system [75]. Immature DCs (with lower levels of HLA-DR, CD80, CD83, and CD86), including bone-marrow-derived immunosuppressive cells [76], could induce T-cell tolerance, whereas mature DCs induce T-cell immunity [77,78,79,80,81]. Immature DCs were found to be associated with longer allograft survival compared with normal controls during the immune response process of corneal transplantation (Figure 2A) [45]. 

Another important type of APC, LCs are bone marrow-derived tissue-resident macrophages [82]. During immune and inflammatory reactions after corneal injury, LCs migrate into the central corneal epithelium along with neutrophils and monocytes [83,84] and can induce cytotoxic T-cell responses towards donor MHC alloantigens in corneal grafts [85]. 

Yamaguchi et al. [86] found a notable up-regulation of the complement activation pathway in corneal transplantation Füst et al. [87] reported that the complement system might be activated both through the classical and alternative pathways in the aqueous humor of the patients with Fuchs’ dystrophy. In addition to this, certain scholars have found that the presence of an increased concentration of C1rs-C1inh complex in tear samples [88], suggests the classical pathway of complement might be activated in the early postoperative period after penetrating keratoplasty. Previously, Zhang X et al. [89] reported that anti-CD45 antibody plus complement-mediated targeting of donor tissue is the most efficient way to deplete corneal passenger leukocytes. More recently, by applying eculizumab, a C5-blockade agent, Islam R et al. [90] introduced a potential therapy for inhibiting xenograft-induced innate inflammatory responses. Despite lacking relevant clinical studies, these data give us new ideas for high-risk corneal transplantation immunotherapeutic strategy.

CCR7 is mainly expressed in activated B and T lymphocytes and can stimulate DC maturation and T-cell homing [91]. The chemokine CCL19 is one of the ligands (the other is CCL21) for CCR7. CCR7 can induce the movement of antigen-specific effector and central memory T cells move to the lymph nodes, followed by CCR7–CCL19 interaction [92]. CCR7-deficient mice show severely delayed kinetics for the antibody response, lack of contact sensitivity, and delayed-type hypersensitivity reactions [93]. Figure 4C shows that blockade of the CCR7–CCL19 axis could prolong mouse corneal-allograft survival. Thus, this blockade could be a potential approach for the manipulation of DC maturation during corneal transplantation. 

During secondary signal transduction, the engagement of T cells and APCs depends on the interaction of CD40, which is expressed by B cells and APCs, with the CD40 ligand (CD40L, also known as CD154), which is expressed by activated T cells [94]. CD40 is a 48 kDa transmembrane protein that is initially expressed on B cells, DCs, macrophages, and monocytes, as well as predominantly on corneal limbal epithelial cells [95,96]. CD154 could be a transmembrane protein or in its soluble form [97]. The engagement of CD40 on the DC surface can promote cytokine production, costimulatory molecule expression, and antigen presentation [98]. However, the inhibition of CD40-CD154 could suppress the secondary signal, consequently reducing allograft rejection, which is consistent with the meta-analysis results in this study (Figure 4B).

Another well-known co-stimulatory molecule pathway is the interaction between the B7 (CD80/86) family and CTLA-4. CTLA-4 is a receptor expressed by both CD4+ and CD8+ T cells, which mediates the suppression of T-cell activation. CTLA-4 interacts with CD80 and CD86 on APCs with high affinity and competes with CD28; its interaction with CD80/86 could induce co-stimulation [99]. Experimental data from articles reviewed here showed that CTLA-4 blockade could improve allograft survival (Figure 4A). Therefore, CTLA-4 could be used in an immune regulatory mechanism to inhibit the ability of APCs to stimulate naïve T cells. 

Recently, owing to its immunosuppressive and tolerogenic properties, programmed death ligand-1 (PD-L1) has drawn scientists’ attention as a potential therapeutic target in corneal transplantation. Nosov et al. [100] reported that local PD-L1 gene transfer in cultured corneas prolonged corneal allograft survival and attenuated graft rejection. Hori et al. [8] suggested that PD-L1-induced apoptosis is a mechanism of immune privilege of corneal allografts. Shen et al. [101] emphasized the importance of peripheral tissue-derived PD-L1 in down-regulating local immune responses in allografts. PD-L1 is constitutively expressed on endothelial cells of the cornea, iris–ciliary body, and neural retinal [102]. Sugita et al. reported that human corneal endothelial cells expressing PD-L1 suppress PD-1^+^ T helper 1 cells via a contact-dependent mechanism [103]. Although there have been no clinical trials on the application of this immune checkpoint inhibitor for corneal transplantation, Dutra et al. [104] reported a successful corneal transplant in a patient treated with Nivolumab for metastatic non-small cell lung cancer and suggested that the treatment with anti-PD1 could not be regarded as an absolute contraindication to corneal transplantation.

IL-1 is an important pro-inflammatory cytokine involved in the initiation of both innate and adaptive immune responses [105]. It has two subtypes: IL-1α and IL-1β. The former mediates the early stages of tissue injury, whereas the latter is responsible for a later inflammatory response [106]. IL-1RA is a suppressor that inhibits IL-1-mediated lymphocyte proliferation. Recombinant IL-1RA specifically inhibits IL-1α and IL-1β activities, and its action has been verified in various inflammatory diseases [107]. In this review, recombinant IL-1RA was found to exhibit the same suppressive effect on allograft rejection (Figure 3A). In clinical research, human corneal limbal epithelial cells were cultured on the amniotic membrane stroma and the results showed that the expression of IL-1RA was upregulated [108]. This underlying mechanism offers new ideas for the clinical reduction of ocular surface inflammation after corneal transplantation.

IL-17A is mainly produced by Th17 cells, which are a subset of the CD4+ T cell-derived population [109]. IL-17A is a pro-inflammatory cytokine implicated in the pathogenesis of inflammatory and autoimmune diseases [110,111,112]. Unlike other pro-inflammatory cytokines, such as IL-1, IFN-γ, and IL-6, IL-17A plays a protective role during acute corneal graft survival. In this review, a meta-analysis of two studies demonstrated that IL-17A depletion using an anti-IL-17A antibody could exacerbate corneal-allograft rejection (Figure 3B). However, Yin et al. [113] reported that prophylactic neutralization of IL-17 significantly increased corneal-allograft survival and reversed rejection in the late-term post-engraftment. Other than in the corneal allograft setting, Kwan T et al. [114] reported that IL-17 deficiency or neutralization was protective against graft rejection in a murine kidney allograft model. This suggests that IL-17 has spatiotemporal heterogeneity in corneal transplantation [33,113,115].

Corneal neovascularization is mediated by VEGF [115,116]; VEGF-A combined with VEGF receptor 1 and VEGFR-2 can initiate neovascularization. Furthermore, VEGF plays an important pathogenic role in ocular surface disorders [116,117,118]. In corneal transplantation, neovascularization is the major cause of graft failure, especially in high-risk corneal grafts [73,119,120,121]. Accordingly, a higher graft survival rate was observed on the use of anti-VEGF antibodies or soluble VEGFR molecules to block this pathway (Figure 3C). In clinical practice, anti-VEGF therapies appear to be the most promising approach for corneal neovascularization in grave condition such as high-risk corneal transplantation. Human studies demonstrated that subconjunctival injection of bevacizumab could reduce corneal neovascularization [122,123,124].

Tregs are a subpopulation of a subset of thymus-derived CD4+ T cells that express high levels of IL-2Rα (CD25); they modulate the immune system, maintain a tolerance to self-antigens, and prevent autoimmune diseases [72]. In mice, Tregs express CD25 and the transcriptional regulator forkhead box P3 (FOXP3) [121]. Tregs have been observed to be effective in preventing autoimmune diseases and delaying graft rejection [119,121,125,126,127,128]. Although their suppressive function has been reported in mouse models (Figure 5), the expansion of the Treg population continues to be difficult.

**Figure 6:**
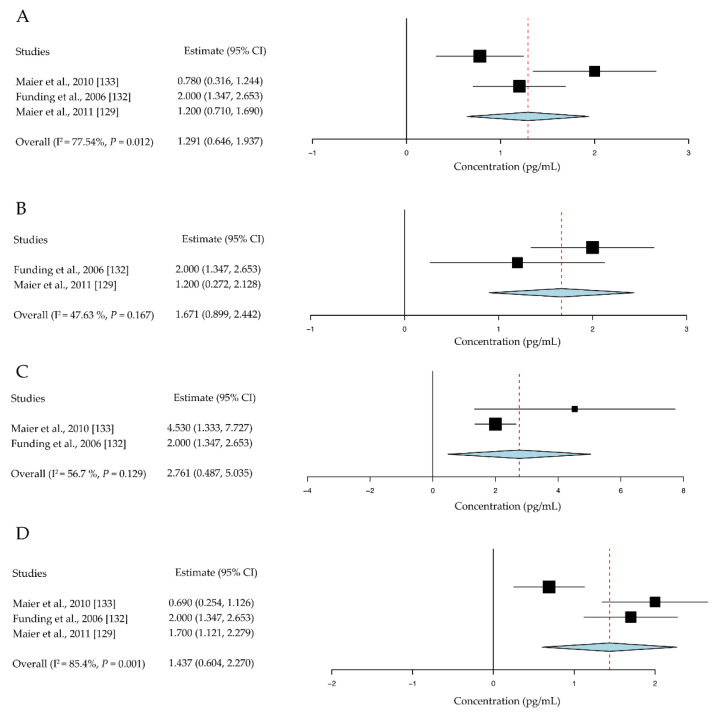
Inflammatory cytokine level in the aqueous humor of human corneal graft cases. (**A**) In the survival group, the level of TNF-α was 1.29 pg/mL (95% CI, 0.65 to 1.94 pg/mL). (**B**) IFN-γ was 1.67 pg/mL (95% CI, 0.90 to 2.44 pg/mL), (**C**) IL-1β was 2.76 pg/mL (95% CI, 0.49 to 5.03 pg/mL), and (**D**) IL-2 was 1.44 pg/mL (95% CI, 0.60 to 2.27 pg/mL). CI, confidence intervals.

There are few clinical trials that use cell therapies or targeted antibodies for prolonging corneal graft survival, demonstrating a large gap between animal research and clinical practice. We collected and analyzed data from clinical reports on inflammatory cytokine levels after human corneal transplantation. Our results showed that TNF-α, IFN-γ, IL-1β, and IL-2 levels in the aqueous humor of keratoplasty rejection cases were much higher than those in cases where the grafts survived [129,130,131,132,133] (Figure 6).

These findings prove that above novel approaches and basic studies conducted in animal models provide promising future perspectives on the application of immunosuppression to improve the outcomes of corneal transplantations. For example, it has been reported that third-party allogeneic mesenchymal stromal cells could prevent rejection in a pre-sensitized high-risk model of corneal transplantation [134]. Moreover, our previous research has revealed the immunoregulatory effect of donor’s bone-marrow-derived suppressor cells in a mouse model of high-risk corneal transplantation [76]. These cellular therapies may provide a potential method of dealing with neovascularization and graft rejection. A phase 1b clinical trial led by VISICORT (EudraCT ID is 2018-000890-60) is currently underway to test the safety and feasibility of healthy donor bone-marrow-derived mesenchymal stem cells as an immunotherapy for patients with a high risk of rejection of corneal transplants. Additionally, clinical studies have suggested that anti-VEGF drugs, including bevacizumab and aflibercept, could be a safe and efficient treatment alternative to improve the outcomes of corneal transplantation in patients. Previous reports have demonstrated that anti-VEGF therapy improved graft survival in patients who have undergone penetrating keratoplasty [135,136,137]. However, when considering the current immunotherapies for corneal transplantation, the diverse experimental methods in animal models and the lack of clinical trials contribute to the disconnection between basic research and clinical practice. Clinical trials using novel therapeutic strategies are required.

**Figure 7:**
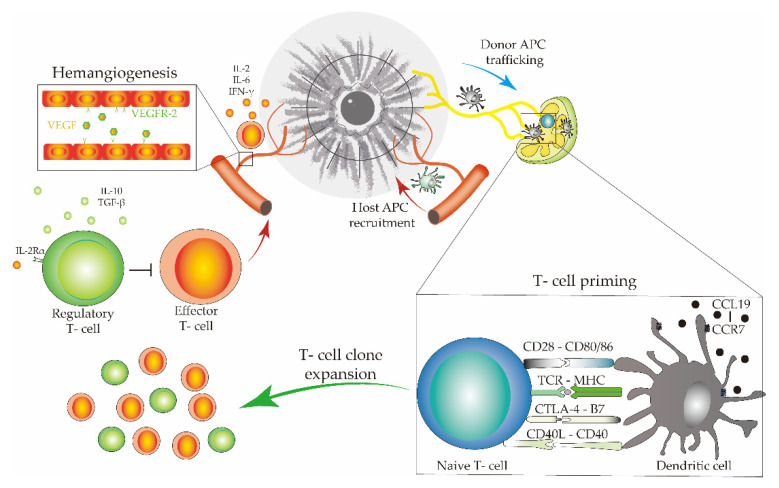
Cells, cell membrane receptors, ligands, and cytokines form the immune pathway involved in corneal graft survival.

Finally, as in other tissues beyond the eye, the homeostatic equilibrium of the ocular surface is critically maintained by complex and heterogeneous parainflammatory mechanisms [138,139,140]. Cross-talk between APCs, T cells, and various other immune cells plays a central role in corneal graft tolerance (Figure 7). Furthermore, graft retention or graft rejection is not always directly related to immune privilege. Conditions such as trauma, infection, and endothelial corneal dystrophy could be the direct cause of corneal graft failure, altering the parainflammatory homeostatic equilibrium.

This study had certain limitations. Firstly, owing to a lack of data from clinical studies, all selected studies were based on murine allogeneic transplantation models, not humans. Second, studies were selected from the same research groups or had similar publication dates because of the small number of included studies. Third, data availability bias may exist because only graft survival was selected as an outcome, and several studies were excluded because of insufficient survival data for analysis. In addition, these reviews were limited to the penetrating keratoplasty models; other surgeries like Descemet-stripping automated endothelial keratoplasty and Descemet-membrane endothelial keratoplasty were not included. The goal of this study was to summarize a wide range of immunotherapy for corneal transplantation; it was difficult to strictly control various biases. Thus, to address these limitations, further studies are required.

## 5. Conclusions

This review demonstrates that the heterogeneity and diversity of immune response in allograft rejection, which may unveil alternative critical and effective targets for improving corneal transplantation survival.

## Figures and Tables

**Table 1:** Inclusion and exclusion criteria for meta-analysis.

**Inclusion criteria**
Study objective: corneal survival or rejection data from murine allogeneic corneal transplantation experiment; various intervention factors were investigated
Study design: experimental research using the murine (mice or rat) corneal allograft model, studied using the valid data of experimental and control groups
Outcome: evaluation of allograft survival from innate and adaptive immunity perspective, case number, mean survival or rejection days with standard deviation, survival or rejection rate, and follow-up duration
**Exclusion criteria**
Experimental methods and protocols, reviews, systematic reviews, and conference proceedings
Studies only involving syngeneic transplantation; allogeneic models with animals other than mice or rats and the xenograft animal model
Preprinted articles
Conference abstracts

**Table 2:** Characteristics of studies included in the meta-analysis.

Source	Publication Date	Country	Species and Allograft Model	No. (Treated/Control)	Intervention Factors	Intervention Method	Evaluation Parameter	Follow-Up Duration
Gao et al. [43]	18 June 2013	China	Rats DA into F344	16/16	Immature DCs	Tail vein	Mean survival days	All rejected on day 25
Yan et al. [44]	14 March 2014	China	Mouse BALB/c into B6	10/10	Bone marrow derived DCs	Tail vein	Mean survival days	8 weeks
Li et al. [45]	27 March 2014	China	Rats SD into Wistar	6/7	Bone marrow derived DCs	Intravenous	Mean survival days	Not mentioned
Callanan et al. [27]	February 1988	United States	Rats Wistar into LEW	23/40	LCs	LCs-containing graft	Rejection rate	>6 weeks
He et al. [28]	January 1996	United States	Mouse NZB into CB6F1	10/21	LCs	LCs-containing graft	Rejection rate	8 weeks
Ross et al. [26]	November 1991	United States	Rats LEW into F344	29/23	LCs	LCs-containing graft	Rejection rate	>6 weeks
Hoffmann et al. [47]	August 1997	Germany	Mouse C3H into BALB/c	5/10	The recombinant fusion protein, CTLA4-Ig	Intraperitoneal injection	Mean rejection days	All rejected on day 24
Zhang et al. [48]	March 2002	Germany	Mouse C3H into BALB/c	6/6	The recombinant fusion protein, CTLA4-Ig	Intraperitoneal injection	Immunoreaction days	40 days
Gong et al. [49]	April 2006	Germany	Rats DA into LEW	6/16	Adenovirus type 5 encoding CTLA-Ig	Intraperitoneal injection	Mean survival days	40 days
Chen et al. [40]	January 2008	China	Mouse B6 into BALB/c	30/30	CTLA4-FasL protein	Protein-immersed graft	Mean survival days	Not mentioned
Qian et al. [29]	April 2001	United States	Mouse B10.D2 into BALB/c	48/48	Anti-CD154 antibody	Intraperitoneal injection	Survival rate	8 weeks
Qian et al. [30]	August 2002	United States	Mouse B10.D2 into BALB/c	10/10	Anti-CD154 antibody	Subconjunctival injection	Survival rate	8 weeks
Jin et al. [32]	February 2010	United States	Mouse B6 into BALB/c	16/16	CCR7 (-/-)	Gene knock-out donor grafts	Survival rate	8 weeks
Hos et al. [53]	May 2016	Germany	Mouse B6 into BALB/c	13/13	CCL19-Ig	Eye drops	Survival rate	8 weeks
Zhou et al. [37]	May 2004	China	Rats Wistar into SD	10/10	IL-1RA	Eye drops	Mean survival days	4 weeks
Jie et al. [38]	May 2004	China	Rats SD into Wistar	10/10	IL-1RA	Eye drops	Mean survival days	Until all rejected
Jie et al. [39]	December 2004	China	Rats F344 into LEW	8/10	IL-1RA	Subconjunctival injection	Mean survival days	Until all rejected
Yuan et al. [42]	May 2013	China	Rats SD into Wistar	20/20	IL-1RA	Subconjunctival injection	Mean rejection days	Until all rejected
Torres et al. [55]	18 August 1998	Portugal	Rats PVG into AO	7/6	Interleukin-10	Subconjunctival injection	Mean rejection days	Until all rejected
Gong et al. [50]	9 November 2006	Germany	Rats Wistar to LEW	6/9	Interleukin-10	IL-10 gene transfer graft	Mean rejection days	Until all rejected
Cunnusamy et al. [33]	15 October 2010	United States	Mouse B6 into BALB/c	10/10	Anti-IL-17A antibody	Intraperitoneal injection	Mean rejection days	8 weeks
Cunnusamy et al. [34]	15 June 2011	United States	Mouse B6 into BALB/c	10/10	Anti-IL-17A antibody	Intraperitoneal injection	Mean rejection days	8 weeks
He et al. [41]	November 2010	China	Mouse B6 into BALB/c	10/10	CD4^+^CD25^+^ T cells	Retroorbital injection	Median survival days	All rejected on day 29
Xu et al. [46]	15 November 2015	China	Mouse B6 into BALB/c	6/6	TGF-β-induced regulatory T cells	Subconjunctival injection	Mean survival days	8 weeks
Cursiefen et al. [31]	August 2004	United States	Mouse B6 into BALB/c	22/22	Molecular trap for VEGF-A	Intraperitoneal injection	Survival rate	8 weeks
Hos et al. [51]	May 2008	Germany	Mouse B6 into BALB/c	11/11	VEGFR-Tyrosine kinase inhibitor	Intraperitoneal injection	Survival rate	8 weeks
Bachmann et al. [52]	August 2009	Germany	Mouse B6 into BALB/c	11/11	Molecular trap for VEGF-A	Intraperitoneal injection	Survival rate	8 weeks
Rocher et al. [54]	January 2011	France	Rats BN into LEW	6/6	Anti-VEGF antibody	Subconjunctival injection	Survival rate	Day 21 *
Cho et al. [35]	December 2012	United States	Mouse B6 into BALB/c	16/11	VEGR-1-morpholino	Subconjunctival injection	Survival rate	8 weeks
Emami-Naeini et al. [36]	November 2014	United States	Mouse B6 into BALB/c	16/10	Soluble VEGFR-3	Intraperitoneal injection	Survival rate	8 weeks

BN, Brown Norway; C57BL/6, B6; DA, Dark Agouti; DC, dendritic cell; F344, Fischer 344; IL-1RA, interleukin-1 receptor antagonist; LC, Langerhans cell; LEW, Lewis; SD, Sprague-Dawley; VEGFR, vascular endothelial growth factor receptor; * stopped observation when all grafts in the control group were rejected and the graft survival score was evaluated experimentally.

## Data Availability

All data generated or analyzed during this study are included in this published article.
